# 

**Published:** 1897-11

**Authors:** 


					﻿Vol. XVII.
NOVEMBER, 1897.
NO. 11.
JPHE
HOMEOPATHIC PHYSICIAN,
A Monthly Journal of Homœopathic Materia Medica
and Clinical Medicine.
"He i* a freeman whom truth makes free, and all are slaves beside.”
"Seek the Truth: come whence it may, cost what it will.”
EDITED AND PUBLISHED BY
WALTER M. JAMES, M. D.
PHILADELPHIA:
ZLTo. 1231 LOCUST STREET.
LONBON:
ALFRED HEATH & CO., Sole Agents fob Gbeat Bbitain,
114 Ebury Street, Eaton Square,
FeorZ}/ Subscription, $2.50, invariably in advance. Single numbers, 25 cts.
Entered at the Philadelphia Past-Office aa Seoond-Claa« Mail Matter.
				

